# Bibliometric and visual analysis of intestinal ischemia reperfusion from 2004 to 2022

**DOI:** 10.3389/fmed.2022.963104

**Published:** 2022-08-15

**Authors:** Yantong Wan, Peng Dong, Xiaobing Zhu, Yuqiong Lei, Junyi Shen, Weifeng Liu, Kexuan Liu, Xiyang Zhang

**Affiliations:** ^1^College of Anesthesiology, Southern Medical University, Guangzhou, China; ^2^Department of Anesthesiology, Hospital of Traditional Chinese Medicine of Zhongshan City, Zhongshan, China; ^3^Department of Anesthesiology, Nan Fang Hospital, Southern Medical University, Guangzhou, China; ^4^The Second Clinical Medical College, Southern Medical University, Guangzhou, China

**Keywords:** intestinal ischemia reperfusion, VOSviewer, CiteSpace, visual analysis, bibliometric

## Abstract

**Background:**

Intestinal ischemia/reperfusion (I/R) injury is a common tissue-organ damage occurring in surgical practice. This study aims to comprehensively review the collaboration and impact of countries, institutions, authors, subject areas, journals, keywords, and critical literature on intestinal I/R injury from a bibliometric perspective, and to assess the evolution of clustering of knowledge structures and identify hot trends and emerging topics.

**Methods:**

Articles and reviews related to intestinal I/R were retrieved through subject search from Web of Science Core Collection. Bibliometric analyses were conducted on Excel 365, CiteSpace, VOSviewer, and Bibliometrix (R-Tool of R-Studio).

**Results:**

A total of 1069 articles and reviews were included from 2004 to 2022. The number of articles on intestinal I/R injury gradually plateaued, but the number of citations increased. These publications were mainly from 985 institutions in 46 countries, led by China and the United States. Liu Kx published the most papers, while Chiu Cj had the largest number of co-citations. Analysis of the journals with the most outputs showed that most journals focused on surgical sciences, cell biology, and immunology. Macroscopic sketch and microscopic characterization of the entire knowledge domain were achieved through co-citation analysis. The roles of cell death, exosomes, intestinal flora, and anesthetics in intestinal I/R injury are the current and developing research focuses. The keywords “dexmedetomidine”, “proliferation”, and “ferroptosis” may also become new trends and focus of future research.

**Conclusion:**

This study comprehensively reviews the research on intestinal I/R injury using bibliometric and visualization methods, and will help scholars better understand the dynamic evolution of intestinal I/R injury and provide directions for future research.

## Introduction

Intestinal ischemic injury occurs in various situations during clinical practice, such as arterial embolism, strangulated hernia, colon cancer, intestinal torsion, blood poisoning, mesenteric dysfunction, and hypovolemic shock (1). During the interruption of blood supply, mitochondrial dysfunction and metabolic disturbances of energy deficiency damage intestinal cells (2, 3); the end of ischemia is often accompanied by tissue reperfusion, which further aggravates intestinal damage (4).

This process is usually accompanied by an intense inflammatory response and massive neutrophil recruitment (5). The release of inflammatory factors and the recruitment of neutrophils cause extensive intestinal epithelial cell death (6), resulting in impaired intestinal mucosal barrier function, which involves distant organs such as lungs, liver, and kidneys (7–9). Consequently, bacteria and endotoxins from the intestinal lumen can transit through the damaged intestinal mucosal barrier to the circulatory system (10), inducing systemic inflammatory response syndrome or even multi-organ failure (11). Microcirculatory disorders and organ damage after intestinal I/R are complex pathological processes involving metabolic damage and oxidative stress (2). Metabolic damage is mainly manifested in ischemia, where vascular closure or obstruction leads to intracellular hypoxia, impairing the expression of mitochondrial respiratory chain ATP synthase and thus resulting in reduced ATP synthesis and ATP deficiency (12, 13). At the same time, the microcirculatory dysfunction caused by I/R injury may initiate multi-organ damage, organ fibrosis, and even organ failure (14). Hence, prevention and treatment of intestinal I/R injury are crucial, given its potential to cause severe deterioration of the patient’s physiological status and its high incidence and mortality in surgical applications.

With the rapid advances in basic medicine of cell death (15), intestinal flora (16), microRNA (17), and various omics (18, 19), many academic journals have published articles on preventing or reducing intestinal I/R injury. However, few attempts have been made to systematically analyze the scientific results and current status of this field from a global perspective. Therefore, a suitable visualization method is urgently needed to reveal the global status, future trends, and hot spots of intestinal I/R injury research.

Since the emergence of the bibliometric field in the 1960s, the open science movement is considered the most critical change in bibliometrics. The free sharing of various scientific results on the Web has influenced the practice of bibliometrics at all levels, including data, infrastructure, definition, and collection of metrics. Bibliometric methods can be used to explore the impacts of the research fields, researchers, and specific papers, or to identify documents that are particularly influential in each research field (20–22). Recently, the results of bibliometric analysis have been applied to medical fields such as cancer (23), stroke (24), critical care medicine (25), and anesthesiology (26). However, there is still a gap in the bibliometric studies on intestinal I/R injury. Therefore, this review systematically analyzes the studies on intestinal I/R injury to assess the current status and hot spots of this field.

## Materials and methods

### Data sources

Our bibliometric analysis is based on the Web of Science Core Collection (WOSCC), the most widely-used and convincing dataset for bibliometrics today (27, 28). WOSCC has the most comprehensive set of data fields to support our most comprehensive analysis. We used “intestinal ischemia reperfusion” (Topic) OR “intestinal ischaemia reperfusion” (Topic) OR “mesenteric ischaemia reperfusion”(Topic) as a search tool to retrieve literature. The categories included 1010 papers, 59 reviews, 46 conference papers, 4 online publications, and 1 data paper. We limited our search to papers and reviews, resulting in 1069 documents. All of the above documents were in English.

### Data collection

All results were searched on WOSCC using the above formula and exported as plain text literature in formats txt and csv. The literature search was conducted on May 1, 2021 to prevent possible bias introduced by database updating.

### Data analysis

Visual analysis was performed on Microsoft Excel 365, Bibliometrix (R-Tool of R-Studio) (29), VOSviewer (30), and CiteSpace (31). The advantages and disadvantages of the above econometric analysis software were also discussed in recent literature, and thus were not elaborated here (32). CiteSpace 6.1.R2 Advanced was used to visually analyze country distribution, institution distribution, subject area distribution, keyword timeline, references, keywords, and literature bursts from the above-extracted data. On VOSviewer 1.6.18, we visually analyzed country distribution, institution distribution, and author distribution from the extracted data. With Bibliometrix (R-Tool of R-Studio), we visually analyzed country distribution, references, and keywords using R-Studio.

Since all raw data used here were obtained from public databases, no ethical review was required.

## Results

### Publication output and temporal trend

**FIGURE 1:**
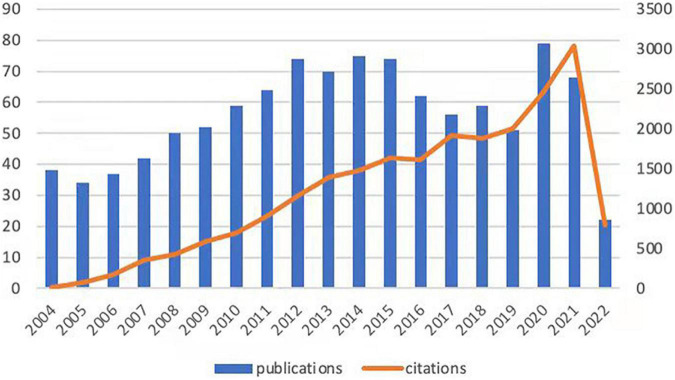
Trends in the publication and citation frequency of intestinal I/R-related literature (2004–2022). The number of publications fluctuates between 30 and 80, with a maximum value in 2020 and an average of more than 66 in the last decade. The overall upward trend in citation frequency is observed.

As a complex pathophysiological process, intestinal I/R has been studied since the second half of the last century (33). Given that the object of analysis should be current and cutting-edge, we set the publication time to be about 19 years or since 2004. The number of articles per year published during the selected period was variable, fluctuating from 30 to 80 (Figure 1). The annual citation frequency in this field generally rose over the past 20 years, with 2,470 citations in 2020, which was one-third more than that in 2019. The annual number of articles and the frequency of citations within the 20 years peaked to 79 documents in 2020 and 3,038 citations in 2021 respectively.

### Distributions of countries/regions and institutions

**FIGURE 2:**
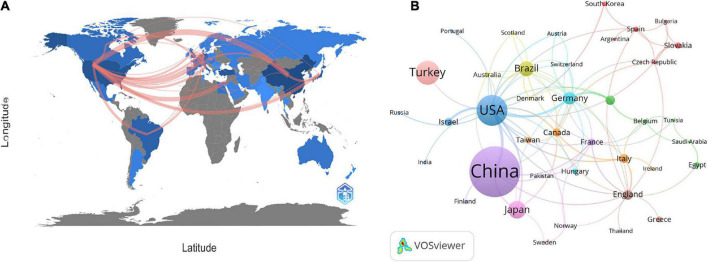
**(A)** Countries or regions involved in the study of intestinal I/R. **(B)** Visualization diagram of each country/region regarding intestinal I/R. Applying VOSviewer, the collaborative network of countries/regions. Different nodes represent different countries or regions. Different colors represent clusters with different affinities. Publications of countries was shown in the sizes of nodes. The ten sets are marked in different colors: China, Finland, France, Pakistan (purple); United States, Israel, Russia, India, Portugal (blue); Germany, Switzerland, Austria, Hungary (light blue); Brazil, Australia, Scotland, Denmark (yellow); Canada, Taiwan, Italy, Iceland (orange); England, Greece, Thailand (brown); Netherlands, Belgium, Tunisia, Saudi Arabia, Egypt (green); Japan, Sweden, Norway (pink); Spain, Slovakia, Korea, Bulgaria, Czechia, and Argentina (red).

At present, 46 countries or regions are involved in intestinal I/R research, which are mainly from the northern hemisphere. There are only Brazil, Argentina, Australia, and New Zealand studied intestinal I/R in the southern hemisphere. The proportion approximately is less than 10% (4/46).What’s more, the links between countries is concentrated in the northern hemisphere. Brazil in the southern hemisphere is relatively active and maintains a high frequency of communication with Europe and the United States (Figure 2A). China had the most significant number of publications with 414 (34.33%), followed by the United States with 184 (15.26%) and Turkey with 126 (10.45%) (Table 1). In terms of total association strength, the United States (88), Germany (33), and China (33) were the strongest and invested more efforts to intestinal I/R research. The top two countries in terms of the number of publications totaled nearly half of the total number. The top ten institutions in terms of literature output are shown in Table 2. Zhongshan University had the most significant number of publications (76, 6.30%), followed by Dalian Medical University (50, 4.15%) and São Paulo University (27, 2.24%). Similarly, Sun Yat-sen University had the strongest total association strength in this field. Meanwhile, Sun Yat-sen University, Technion-Israel Institute of Technology, University of São Paulo, Southern Medical University, Wuhan University, the Federal University of São Paulo, and Ohio State University all rank in the top 10 in terms of the number of articles and association intensity and should have conducted extensive research in this field.

**TABLE 1:** Top 10 countries in terms of number of publications, frequency of citations, and total association intensity.

Rank	Countries	Documents	Countries	Citations	Countries	Total link strength
1	China	414	China	7545	United States	88
2	United States	184	United States	6894	Germany	33
3	Turkey	126	Japan	1956	China	33
4	Japan	75	Turkey	1454	England	21
5	Brazil	58	Germany	1164	France	18
6	Germany	44	Hungary	924	Brazil	16
7	England	30	Brazil	907	Canada	15
8	Netherlands	28	Netherlands	801	Italy	15
9	Italy	27	England	666	Japan	15
10	Canada	22	Canada	529	Netherlands	14

**TABLE 2:** Top 10 institutions in terms of number of articles issued and intensity of association.

Rank	Institution	Publications	Original country	Institution	Total link strength	Original country
1	Sun Yat-sen Univ	76	China	Sun Yat-sen Univ	49	China
2	Dalian Med Univ	50	China	Technion Israel Inst Technol	33	Israel
3	Univ Sao Paulo	27	Brazil	Univ Michigan	27	United States
4	Southern Med Univ	25	China	Harvard Univ	26	United States
5	Wuhan Univ	25	China	Univ Sao Paulo	24	Brazil
6	Second Mil Med Univ	23	China	Southern Med Univ	23	China
7	Univ Fed Sao Paulo	22	Brazil	Wuhan Univ	22	China
8	Ohio State Univ	21	United States	Univ Fed Sao Paulo	21	Brazil
9	Maastricht Univ	19	Netherlands	Univ Hong Kong	21	China
10	Technion Israel Inst Technol	18	Israel	Ohio State Univ	20	United States

**FIGURE 3:**
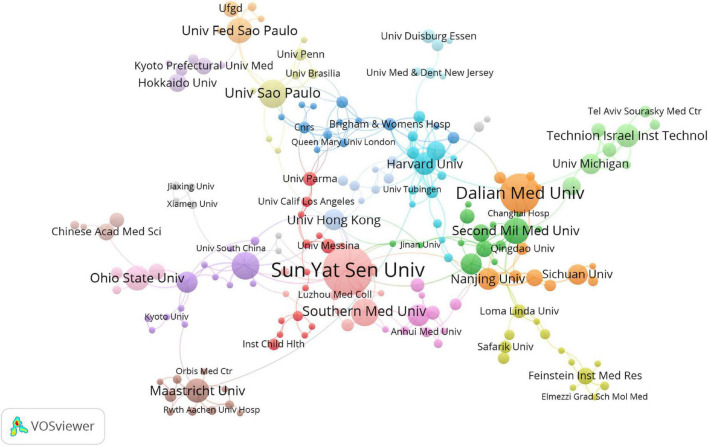
Visualization diagram of each institutions regarding intestinal I/R. Applying VOSviewer, the collaborative network of institutions. Different nodes represent different institutions. Different colors represent clusters with different affinities. Sizes of nodes ars based on publications of institutions.

There is a general lack of cooperation among countries and among institutions in intestinal I/R research, given the limited communication targets, and multiple clearly-differentiated communication scopes in this field. The different colors between nodes analyzed on VOSviewer each indicate a collection of intimacy (30). The countries or regions can be divided into ten sets according to the intimacy of cooperation (Figure 2B). It is a interesting information that, Turkey is a self-contained collection, with only the United States. In contrast, analysis of the country relationship network on CiteSpace showed the periphery of nodes representing the United States, China, England, Germany, Italy, France, and Spain had purple circles in the network, indicating these countries are highly influential in this field (Supplementary Figure 3). These influential countries, except China and France, are located in different groups (Figure 2B). In terms of research focus and collaboration, these high-impact countries may be more likely to differ, which is reflected in the country relationship maps produced on CiteSpace. The relationships between the higher-impact countries do not differ in strength from their relationships with other countries, which shall be anomalous given the volumes of articles published by these high-impact countries. Noticeably, the same mismatch among institutions in terms of volume of issuance and intensity of communication exists in the institutional partnership graph analyzed on CiteSpace, with no relationship between the intensity of each node and other factors. The cooperation among institutions is shown in Figure 3, where 19 sets can be counted. In the light red set, Sun Yat-sen University is closely associated with Southern Medical University, and Luzhou Medical College. In the sky-blue set, Harvard University has close communication with Univ. Tubingen. Other main sets are Brigham & Womens Hosp, Queen Mary Univ. London, CNRS (dark blue); Dalian Medical University, Nanjing University, and Sichuan University (orange), which may have cooperation; Second Military Medical University, Shanghai Jiao Tong Univ., Tongji Univ. (green); Israel Institute of Technology, Univ. Michigan, Tel. Aviv. Sourasky Med. Ctr. (light green); Orbis Med. Ctr and Rwth Aachen Univ. Hosp, Maastricht Univ. (brown), which lack external links. There are also several sets with only a single link to the outside world, such as the light brown set of Chinese Academy of Medical Sciences, the gray sets of Xiamen University, and Jiaxing University, Univ Med&Dnet News Jersey and Univ. Duisburg Essen, Hokkaido Univ. and Kyoto Prefectural Univ. Med., and Loma Linda Univ., Safarik Univ. and Feinstein Grad. Sch. Mol. Med., and other yellow sets. Moreover, in CiteSpace, institutions with strong influence do not appear among these institutions, and some institutions with a considerable volume of issuance have almost no connection with other institutions (Supplementary Figure 4).

### Distributions of authors and co-cited authors

**FIGURE 4:**
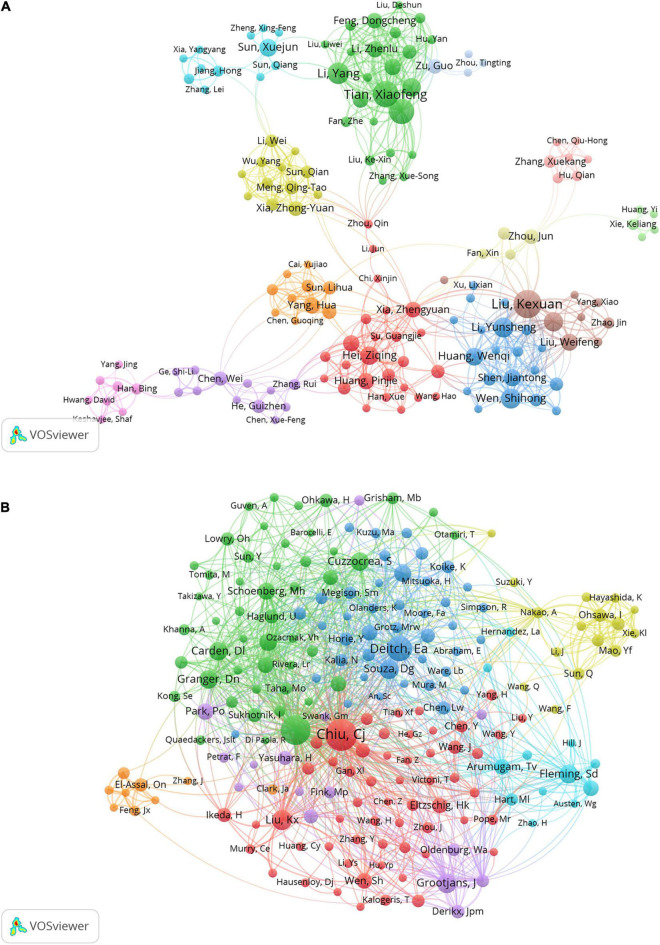
Visualization of association graphs of co-authors and co-cited authors in intestinal I/R. **(A)** Network visualization diagram of collaboration among authors. Sizes of nodes represent publications of authors. Different colors each represent a closer collaborative relationship. The thickness of the inter-author linkage represents the strength of the relationship. **(B)** Network of co-cited authors. Sizes of nodes represent co-citations of authors. Chiu, Cj has the highest number of co-citations.

The authors of the retrieved literature were analyzed, and the top 10 aX2uthors ranked by the number of publications and frequency of citations were found (Table 3). The top 10 authors (except Besner Gail from the United States) were from China, and their institutions were Dalian Medical University, Sun Yat-sen University, and Southern Medical University. The author with the highest number of publications is Kexuan Liu with 35 publications from Southern Medical University, followed by Tian Xiaofeng, and Yao Jihong from Dalian Medical University with 30 and 29 publications respectively. Among the top 10 co-cited authors, Deitch Ea, Carden Dl, and Granger Dn are all from Louisiana State University, United States. Noticeably, most of the results of Mallick Ih, Deitch Ea, and Carden Dl were published before 2004, and were recognized by researchers in the field. In addition, Kexuan Liu appears among the top 10 authors ranked by the number of publications and frequency of citations. This means this author should enjoy a very high level of activity and prestige in the field. The most frequently cited author is Chiu Cj from McGill University, who is not involved in intestinal I/R research and whose research results may be very relevant to the focus on intestinal I/R (34). Figure 4 visualizes the collaborative relationships between authors in the literature related to intestinal I/R, with a specific color representing one affinity (35). Similar to the network of national and institutional relationships, the co-authors form a total of 13 groups (Figure 4A), including a light green group (Xie Keliang, Huang Yi), a light red group (Hu Qian, Zhang Xue-Kang, Chen Qiu-Hong), and a pink group (Han Bing, Yang Jing, Hwang Qiu-Hong). The pink group (Yang Jing, Hwang David) and the light blue group (Zu Guo, Zhou Tingting) are both only cooperatively associated with a single set. The brown group of Liu Ke-Xuan, Li Cai, and Liu Weifeng is cooperatively associated with Wen Shihong. The green group of Tian Xiaofeng, Li Yang, and Yao Jihong has frequent internal connections with the yellow group of Xia Zhong-Yuan, Meng Qing-Tao, and Sun Qian, but lacks connections with the outside world. The red group of Hei Ziqing, Xia Zhengyuan, and Gan Xiaoliang is more extensively associated with the outside world. The different colors in the co-cited author relationship network of Figure 4B partially reflect the same characteristics among the studies of co-cited authors. The focus of attention from the authors of the intestinal I/R literature is highly homogeneous, and only the yellow group (Nakao A, Ohsawa I, Sun Q) and the orange group (El-Assal On, Zhang J, Feng Jx) are sparsely connected to other groups, which can be divided into seven groups in total. Chiu Cj has the highest number of co-citations, followed by Mallick Ih, and Deitch Ea, who enjoy high academic reputation in the field.

**TABLE 3:** Top 10 authors in terms of number of publications and frequency of co-citations.

Rank	Author	Publications	Countries/regions	Institutions	Author	Co-citations	Countries /regions	Institutions
1	Liu, Kexuan	35	China	Nanfang Hospital, Southern Medical University	Chiu, Cj	300	Canada	McGill University
2	Tian, Xiaofeng	30	China	The Second Affiliated Hospital of Dalian Medical University	Mallick, Ih	259	England	Royal Free and University College Medical School
3	Yao, Jihong	29	China	College of Pharmacy, Dalian Medical University	Deitch, Ea	188	United States	Louisiana State University Medical Center
4	Li, Yang	20	China	The Second Affiliated Hospital of Dalian Medical University	Liu, Kx	122	China	Nanfang Hospital, Southern Medical University
5	Li, Yunsheng	18	China	The First Affiliated Hospital of Sun Yat-sen University	Grootjans, J	113	Netherlands	Maastricht University Medical Center, University of Amsterdam
6	Wen, Shihong	18	China	The First Affiliated Hospital of Sun Yat-sen University	Souza, Dg	111	Brazil	Universidade Federal de Minas Gerais
7	Besner, Gail E.	17	United States	Children’s Hospital, The Ohio State University	Cuzzocrea, S	107	Italy	University of Messina
8	Li, Cai	17	China	Nanfang Hospital, Southern Medical University	Carden, Dl	105	United States	Louisiana State University Medical Center
9	Hei, Ziqing	16	China	The Third Affiliated Hospital of Sun Yat-sen University	Granger, Dn	105	United States	Louisiana State University Health Sciences Center
10	Zhang, Feng	16	China	The Second Affiliated Hospital of Dalian Medical University	Fleming, Sd	100	United States	Kansas State University

### Distribution of journals

**FIGURE 5:**
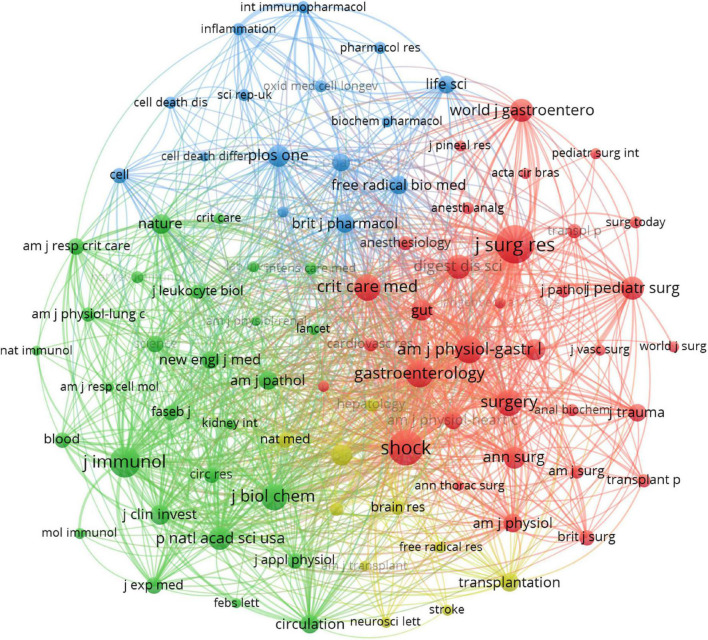
Visualization of the relationship between co-cited journals. One node represents one journal, and the area means the citation frequency. Sizes of nodes are reflected with co-citations.

Journal of Surgical Research (IF 2.192), and Shock (IF 3.454) were the first and second most published and co-cited journals (Table 4). World Journal of Gastroenterology (IF 5.742) was the third most published journal, and Journal of Immunology (IF 5.422) was the third most-cited journal. Six of the top ten journals ranked by the number of publications were also among the top ten journals in terms of co-citation frequency, and these journals should have high prestige in intestinal I/R. The journals with the most publications were 1 in Q1 and 5 in Q2, and the journals with the most co-citations were 3 in Q1 and 5 in Q2 (Table 4), indicating these journals have a high academic reputation in the field. The journals were divided by the co-citation frequency into 4 clusters (Figure 5). Articles from journals within the same cluster are more likely to have similar research directions or a specific internal logic (36). Journal of Surgical Research, and Shock, the top two journals in terms of co-citation frequency, clearly tend to be co-cited with more frequently cited journals, such as World Journal of Gastroenterology, Annals of Surgery, Surgery, and Gut. Immunology has more co-citation potential with Journal of Experimental Medicine, Nature, and American Journal of Pathology.

**TABLE 4:** Top 10 journals in terms of number of issues and co-citation frequency.

Rank	Journal	Publications	% of 1069	IF (JCR 2020)	JCR quatile	Journal	Total co-citations	IF (JCR 2020)	JCR quatile
1	Journal Of Surgical Research	94	8.793	2.192	Q3	J Surg Res	1391	2.192	Q3
2	Shock	31	2.900	3.454	Q2	Shock	1154	3.454	Q2
3	World Journal Of Gastroenterology	29	2.713	5.742	Q2	J Immunol	859	5.422	Q2
4	Plos One	28	2.619	3.24	Q2	Gastroenterology	723	22.682	Q1
5	Transplantation Proceedings	23	2.152	1.066	Q4	Am J Physiol-Gastr L	694	4.052	Q2
6	Journal Of Pediatric Surgery	21	1.964	2.545	Q2	J Biol Chem	647	5.157	Q2
7	Acta Cirurgica Brasileira	19	1.777	1.388	Q4	Crit Care Med	642	7.598	Q1
8	Digestive Diseases And Sciences	18	1.684	3.199	Q3	Surgery	639	3.982	Q1
9	Surgery	17	1.590	3.982	Q1	Digest Dis Sci	549	3.199	Q3
10	Journal Of Immunology	16	1.497	5.422	Q2	J Pediatr Surg	497	2.545	Q2

### Distribution of subject areas

**FIGURE 6:**
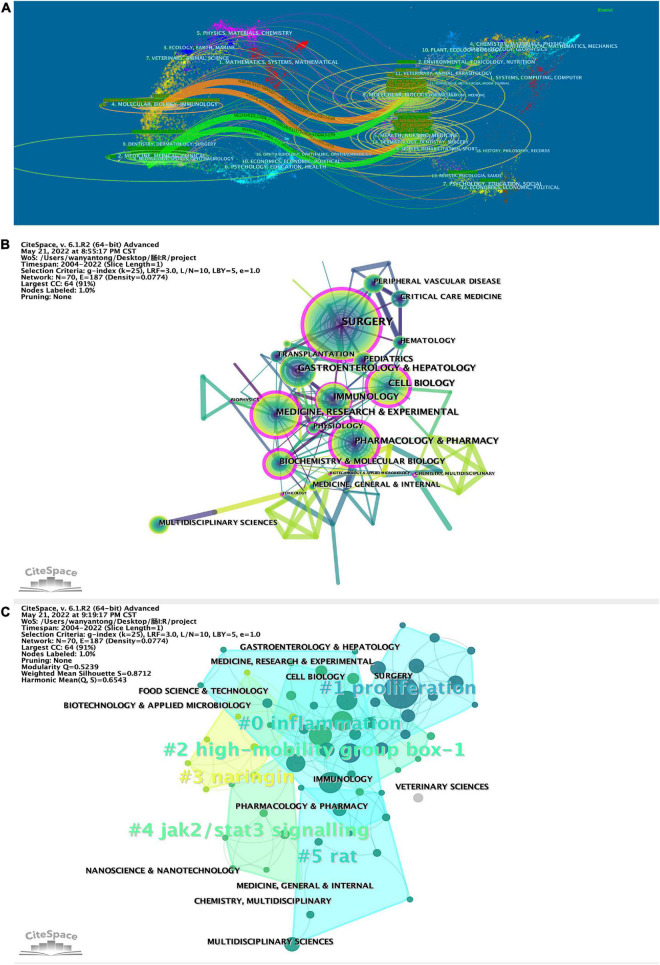
**(A)** Biplot of published and cited literature journals, with published journals on the left and cited journals on the right. CiteSpace was applied to analyze **(B)** the citation relationship between the subject areas in which the literature was published and **(C)** clustered. Sizes of nodes represent publications of subject in **(B)** and **(C)**. The betweenness centrality of a node in the network measures the importance of the position of the node in the network, in **(B)**. Clustering module value (Q value), using in CiteSpace cluster analysis, it is generally considered that Q > 0.3 means that the clustering structure is significant, shown line 10 in top left of **(C)**. The average contour value of clustering (S value), in line 11, it is generally considered that S > 0.5 clustering class is reasonable and S > 0.7 means that the clustering is convincing.

Analyzing the published literature on intestinal I/R and the subject areas of interest enables us to identify the current research habits and scenario applications in intestinal I/R. By tracking the subject areas of the journals published and cited, we linked the visualization in Figure 6A. The colored paths between the citing journals on the left and the cited journals on the right reflect the citation relationship among different fields. The orange and green paths show that the literature on intestinal I/R is mainly published in the disciplinary fields: “4. MOLECULAR, BIOLOGY, IMMUNOLOGY” and “2. MEDICINE, MEDICAL, CLINICAL”. The literature related to both disciplines is more likely to be published in “8. MOLECULAR, BIOLOGY, GENETICS” and “5. HEALTH, NURSING, MEDICINE”. The discussion of intestinal I/R was published or cited in basic medicine, clinical applications, and health care.

Then the literature was analyzed on CiteSpace to refine the citation relationships among subject areas (Figure 6B). The subject “SURGERY” is the most frequently cited. Subjects “SURGERY”, “CELL BIOLOGY”, “IMMUNOLOGY”, “MEDICINE”, “RESEARCH & EXPERIMENTAL”, “PHARMACOLOGY & PHARMACY”, “BIOCHEMISTRY & MOLECULAR BIOLOGY”, “PHYSIOLOGY”, “TOXICOLOGY”, “BIOPHYSICS”, “BIOTECHNOLOGY & APPLIED MICROBIOLOGY”, and “CHEMISTRY, MULTIDISCIPLINARY” are marked with purple circles in the diagram. Results show in the study and research on intestinal I/R, the knowledge of the subjects mentioned above and is even indispensable and shall be given some importance. As shown in Figure 6A, the literature in the subject “IMMUNOLOGY” is mostly referenced by “SURGERY” and “GASTROENTEROLOGY & HEPATOLOGY”. “HEPATOLOGY” and “BIOPHYSICS” are referenced by “MEDICINE, RESEARCH & EXPERIMENTAL”. The literature was clustered by subject areas (Figure 6C) into totally six clusters. The more important clusters are #0, covering “ENDOCRINOLOGY & METABOLISM”, “MICROBIOLOGY”, “GASTROENTEROLOGY & EXPERIMENTAL”, “GASTROENTEROLOGY & HEPATOLOGY”, with the keyword inflammation; #1, covering “TRANSPLANTATION”, “IMMUNOLOGY”, and “SURGERY”, with the keyword proliferation. Interestingly, cluster #3 covers the subject areas “AGRICULTURE, MULTIDISCIPLINARY”, “CHEMISTRY, APPLIED” and “FOOD SCIENCE & TECHNOLOGY”, with the keyword naringin, and may be a collection of disciplines related to the study of components in food. Cluster #5 covers the subject areas “SPORT SCIENCES”, “MEDICAL LABORATORY TECHNOLOGY”, “MEDICINE, GENERAL & INTERNAL”, and “TOXICOLOGY”, and the keyword is rat, which may focus on the animal behavior in intestinal I/R, toxicological response to interventional drugs, and model evaluation.

### Highly cited reference analysis

Therapeutic Effects of Xanthine Oxidase Iinhibitors: Renaissance Half A Century after Discovering Allopurinol (Pacher et al.) (37) had the highest number of citations (843) (Table 5). This paper is based on allopurinol and its active metabolites, which are promising drugs for treating ischemic and other tissue and vascular injuries, inflammatory diseases, and chronic heart failure. This paper is aimed to describe their therapeutic application in various pathophysiological conditions, such as intestinal I/R and to review various possible emerging therapies based on the above approach (37). The second most cited article is In Vitro and In Vivo Antioxidant Properties of Chlorogenic Acid and Caffeic Acid (Stao et al.) (38) with 580 citations. Stao et al. focused on the protective efficacy of chlorogenic acid and the antioxidant activity of caffeic acid when applied to intestinal I/R models (38). Platelet-Derived Stromal Cell-Derived Factor-1 Regulates Adhesion and Promotes Differentiation of Human CD34(+) Cells to Endothelial Progenitor Cells by Stellos et al. (39) was the third most cited article with 233 citations. The authors hypothesized and demonstrated that chemotaxis of platelet-derived stromal cell-derived factor-1 in intestinal I/R promotes ischemic tissues. The underlying mechanisms are related to progenitor cell adhesion and chemotaxis to endothelial cells (39). The fourth most cited article is Mannose-Binding Lectin Is A Regulator of Inflammation that Accompanies Myocardial Ischemia and Reperfusion Injury. With an intestinal I/R model of complement-related gene-deficient mice, this paper showed that mannose-binding lectin (MBL), a circulating pattern recognition molecule, is involved in the aseptic inflammatory response while activating the MBL pathway of complement to damage myocardium, contributing to intestinal I/R. This explains the mechanism of distant organ damage induced by intestinal I/R (40). Most of the previous studies on intestinal I/R are based on the validation of animal and cellular experiments. While the highly-cited articles reflect the high recognition of their authors in the field, the first four highly-cited articles were published over ten years ago, indicating that the research in the last decade is likely to involve the application of tools related to antioxidant, cellular repair and complementation. Hence, the significance of the highly-cited articles should be analyzed considering the confounding of their temporal factors. Within the time scope to the last five years, the top two highly-cited articles both link the focus of intestinal I/R studies to ferroptosis. Ischemia-Induced ACSL4 Activation Contributes to Ferroptosis-Mediated Tissue Injury in Intestinal Ischemia/ Reperfusion reports the possibility of ferroptosis in intestinal I/R at the levels of protein expression and lipid peroxidation (41). Inhibitor of Apoptosis-Stimulating Protein of p53 Inhibits Ferroptosis and Alleviates Intestinal I/R-Induced Acute Lung Injury presents the signaling mechanism how ferroptosis acts with intestinal I/R-induced acute lung injury (42).

**TABLE 5:** Top 10 highly cited references.

Rank	Authors	Article title	Source title	Cited	Year	DOI
1	Pacher et al. (37)	Therapeutic effects of xanthine oxidase inhibitors: Renaissance half a century after the discovery of allopurinol	PHARMACOLOGICAL REVIEWS	843	2006	10.1124/pr.58.1.6
2	Sato et al. (38)	In vitro and in vivo antioxidant properties of chlorogenic acid and caffeic acid	INTERNATIONAL JOURNAL OF PHARMACEUTICS	580	2011	10.1016/j.ijpharm.2010.09.035
3	Stellos et al. (39)	Platelet-derived stromal cell-derived factor-1 regulates adhesion and promotes differentiation of human CD34(+) cells to endothelial progenitor cells	CIRCULATION	233	2008	10.1161/CIRCULATIONAHA.107.714691
4	Walsh et al. (40)	Mannose-binding lectin is a regulator of inflammation that accompanies myocardial ischemia and reperfusion injury	JOURNAL OF IMMUNOLOGY	207	2005	10.4049/jimmunol.175.1.541
5	Harboe et al. (93)	The quantitative role of alternative pathway amplification in classical pathway induced terminal complement activation	CLINICAL AND EXPERIMENTAL IMMUNOLOGY	190	2004	10.1111/j.1365-2249.2004.02627.x
6	Li et al. (41)	Ischemia-induced ACSL4 activation contributes to ferroptosis-mediated tissue injury in intestinal ischemia/reperfusion	CELL DEATH AND DIFFERENTIATION	192	2019	10.1038/s41418-019-0299-4
7	Zhang et al. (94)	Identification of a specific self-reactive IgM antibody that initiates intestinal ischemia/reperfusion injury	PROCEEDINGS OF THE NATIONAL ACADEMY OF SCIENCES OF THE UNITED STATES OF AMERICA	188	2004	10.1073/pnas.0400347101
8	Zhang et al. (95)	Identification of the target self-antigens in reperfusion injury	JOURNAL OF EXPERIMENTAL MEDICINE	175	2006	10.1084/jem.20050390
9	Ohta (96)	Recent Progress Toward Hydrogen Medicine: Potential of Molecular Hydrogen for Preventive and Therapeutic Applications	CURRENT PHARMACEUTICAL DESIGN	177	2011	10.2174/138161211797052664
10	Vollmar et al. (97)	Intestinal ischemia/reperfusion: microcirculatory pathology and functional consequences	LANGENBECKS ARCHIVES OF SURGERY	177	2011	10.1007/s00423-010-0727-x

**FIGURE 7:**
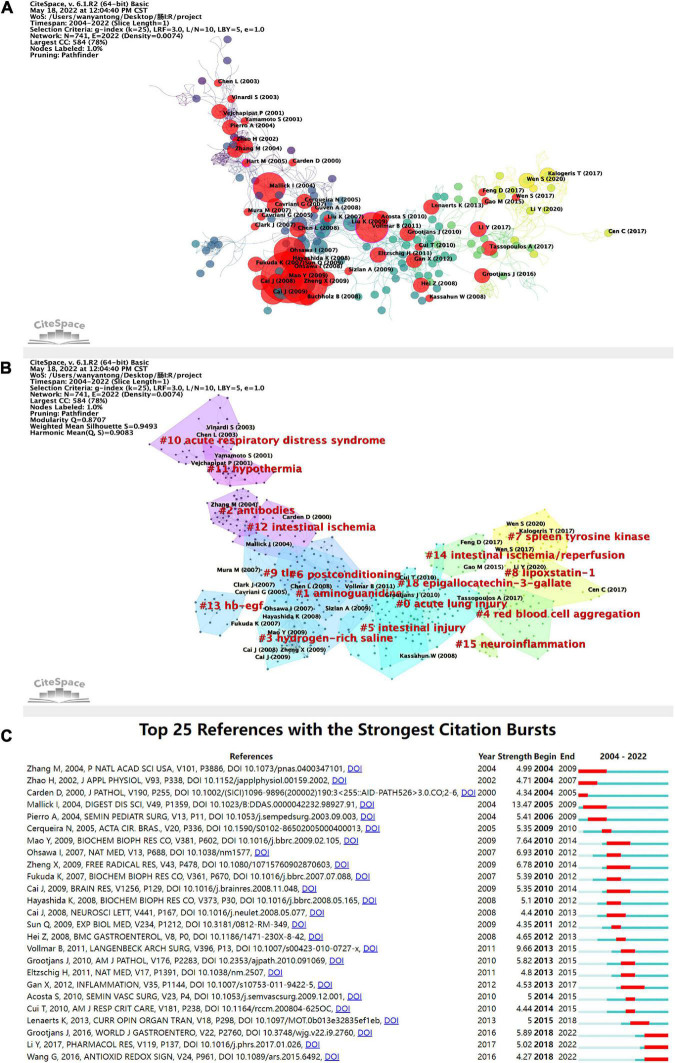
**(A)** Application of CiteSpace, literature relationship network diagram. The different colors of the nodes represent additional years, from purple to yellow, the later the time. In addition, the red nodes represent literature with citation burst. The node size represents the citation frequency. **(B)** Clustering of the literature in the literature relationship network graph, with a total of 17 clusters. **(C)** Top 25 high burst of cited literature. The blue line indicates the timeline, and the red phase represents the outbreak period. It is based on the burst test to detect sudden changes in information like documents and keywords. In CiteSpace, the burst test is developed by Kleinberg J. The meanings of the burst test represent a sharp increase in document citations and some critical questions in the field proposed or solved in the documents.

The relationship among the studies was analyzed on CiteSpace, and was found to have a specific time factor of publication, marking the literature with an explosive citation frequency (Figure 7A). The earliest bursting articles were earlier than 2001. The number of explosive studies and the citation frequency of published studies peaked in 2008. The number of cited incendiary studies is correlated to the frequency of citations of published studies in the same year. The studies with high citation frequency account for a higher percentage among all the high explosive studies. The studies with high citation frequency are more likely to be cited as high incendiary literature, which confirms the correlation between explosive research and citation frequency to a certain extent. It is indicated that the research focus in the field may revolve around a specific hot spot for a while. The clustering of the relational network of the literature (Figure 7B) yielded totally 17 co-reference clusters. The cluster with the highest number of publications is #0, and the specific common keyword with a high number of publications is acute lung injury. The most relevant study is Effect of Montelukast on Acute Lung Injury Induced by Intestinal Cancer by Terzi et al. published in ACTA MEDICA MEDITERRANEA (43). The cluster with more bursts of highly-cited articles is #3, with the keyword hydrogen-rich saline, but this cluster is weakly linked to other clusters. The research in this cluster was probably only a brief hotspot around 2007-2009, with the most relevant literature being Hydrogen Resuscitation, A New Cytoprotective Approach by Zheng Xing-feng et al., who investigated the role and mechanism of hydrogen resuscitation therapy applied with hydrogen-rich saline in intestinal I/R (44). In the statistical index, the cluster with the highest centrality and sigma literature is #0, with the keyword acute lung injury, indicating the high impact topic in this field around 2010 is the acute lung injury in intestinal I/R.

The annual citation frequency has a time burst in the literature somehow with a particular direction in the field to get concentrated attention, which can reflect the emerging academic trends and new topics, predict the frontier direction, and reveal the potential hot spots in a field. The top 25 cited outbreaks shown in Figure 7C started 1-5 years after publication, with three episodes beginning in 2004, and most of them concentrated from 2009 to 2015. There have been three high outbreaks since 2018. Based on their contents, it can be assumed that the current research focus in this field includes the exploration of more effective intestinal I/R models and the application of exosome inhibition and intestinal barriers (45–47).

### Keyword analysis

As a core summary of article contents, keywords can be used to analyze the frontiers of results in intestinal I/R (48). The keywords that appeared more than 100 times in the order of frequency were ischemia/reperfusion (349 times), intestinal I/R (218 times), intestine (208 times), oxidative stress (144 times), inflammation (100 times), and five others (Table 6). The five keywords also have high association strength in the domain (all greater than 1500). Among the top 20 keywords, it is tentatively speculated to have more apparent connections. For instance, ischemia/reperfusion, intestinal I/R, ischemia, and intestinal ischemia all involve the description of intestinal I/R and its related or similar pathophysiological processes; intestine, lung injury, intestinal injury, and injury may focus on the organs affected by intestinal I/R and the resulting injuries. Other keywords are oxidative stress, antioxidants, nitric-oxide, neutrophil, and inflammation with cytokine and NF-κb. Similar relationships may exist.

**TABLE 6:** Top 20 keywords in terms of frequency of occurrence.

Rank	Keyword	Occurrences	Total link strength	Rank	Keyword	Occurrences	Total link strength
1	ischemia/reperfusion	349	732	11	cytokine	40	107
2	intestinal I/R	218	352	12	nitric oxide	25	62
3	Intestine	208	485	13	antioxidant	22	66
4	oxidative stress	144	372	14	lung	20	66
5	inflammation	100	250	15	neutrophil	20	50
6	apoptosis	80	214	16	injury	18	54
7	lung injury	80	206	17	mast cell	17	33
8	Ischemia	76	221	18	nf-kappa b	17	41
9	Rat	68	192	19	bacterial translocation	16	32
10	intestinal ischemia	46	75	20	intestinal injury	16	30

**FIGURE 8:**
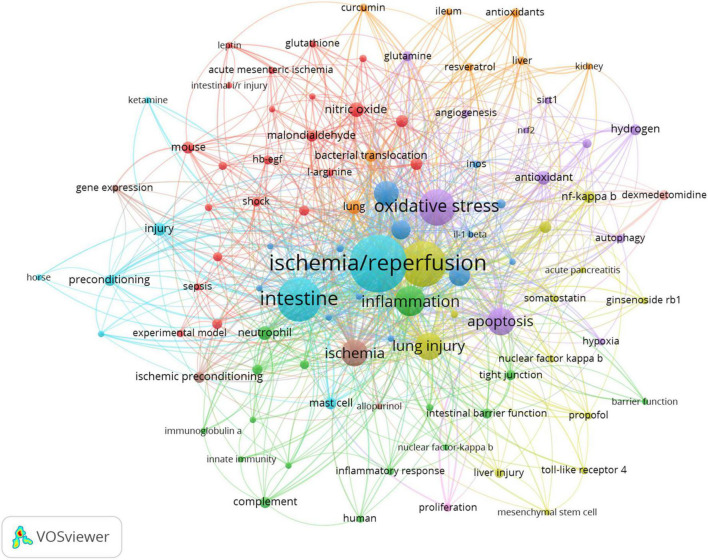
Keyword co-occurrence network relationship diagram. Ischemia/reperfusion has most co-occurrence with others keywords.

Figure 8 shows the network relationship diagram of keyword co-occurrence. The thicker line between the keyword points in each section means the more frequent co-occurrence (48). The different colors of the nodes represent different clusters, and the intestinal I/R field is co-categorized into 10 clusters, reflecting the possible ten research directions. These clusters, with apparent associations among the top 20 high-occurrence keywords, are in general agreement. In the purple cluster, oxidative stress and antioxidant are solidly related with apoptosis, autophagy, and related pathways such as nrf2 and sirt1. In the green cluster, inflammation is associated with neutrophil, immune-related concepts such as complement, and barrier-related concepts such as tight junction. The yellow cluster covers injury, lung injury, liver injury, acute pancreatitis, and other organ injury-related keywords. The orange cluster includes organs such as lung, liver, curcumin, ileum and kidney. In the cyan cluster, ischemia/reperfusion, intestine, and injury show no more obvious association, and are too closely connected to other collections, presumably because it is the standard part of the research direction of the field. In the red cluster, mouse, nitric oxide, experimental model, shock, and sepsis may describe the current situation of intestinal I/R on sepsis and shock research. The brown cluster covers gene expression. The pink cluster (proliferation), and the scarlet cluster (dexmedetomidine) seem to have less number of keywords, which may be emerging research directions.

**FIGURE 9:**
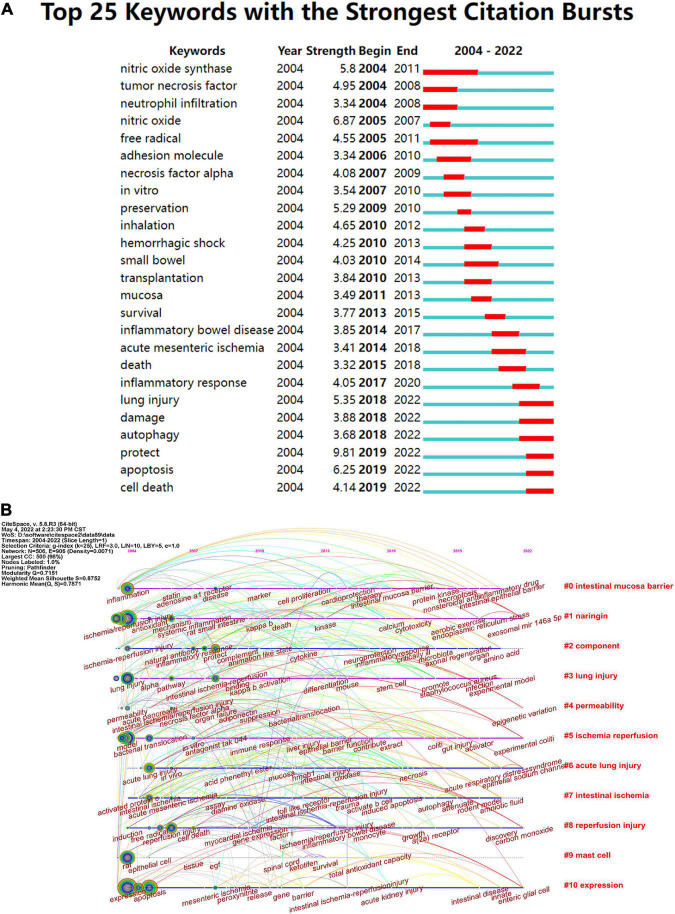
**(A)** Top 25 keywords with the strongest citation frequency burst. **(B)** Visualization map of timeline viewer related to intestinal I/R produced by CiteSpace, expanding the literature clustering by timeline and marking the first occurrence time of each critical keyword.

The keyword burst can also reflect some emerging academic trends and new topics in the field, which can be used to predict the direction of cutting-edge research and reveal potential hot spots in the field (49). The top 25 keywords with high citation bursts in intestinal I/R are shown in Figure 9A. The highest keyword is “protect”, followed by “nitric oxide” and “apoptosis”. “Nitric oxide synthase” had the longest burst time and was the earliest burst among the top 25 keywords. Conversely, “preservation”, “necrosis factor alpha”, “nitric oxide” and “mucosa” all had shorter outbreaks. The keywords that are still in the burst status are “lung injury”, “damage”, “autophagy”, “protection”, “mucosa”, “protect”, “apoptosis” and “cell death”, which represent some of the current hotspots in the field.

Timeline views of the intestinal I/R co-citation network were made with CiteSpace (Figure 9B), which can be seen as emergence, popularity, and decline of hotspots (50). From 2004 to 2010, the strong influence keywords were focused, and were mainly inflammation, ischemia/reperfusion injury, lung injury, model, acute lung injury, activated protein c, rat, expression, reperfusion injury, protect, disease, toll-like receptor, myocardial ischemia, mesenteric ischemia, and immune response. Cluster #1 arose first with the keyword ischemia/reperfusion injury, showing a discovery history in mechanism and countermeasure of I/R injury taken under systemic inflammation and oxidative stress. Cluster #0 (intestinal mucosa barrier), Cluster #1 (naringin), Cluster #5 (ischemia/reperfusion), Cluster #6 (acute lung injury), and Cluster# 10 (expression) were all immediate parts of the research focus.

## Discussion

CiteSpace 6.1.R2 Advanced, VOSviewer 1.6.18, and R-bibliometrix were used to analyze the data of 1069 articles on intestinal I/R between 2004 and 2022 from the Web of Science, and evaluate the spatial and temporal distributions, author contributions, core articles, research hotspots and frontiers of the field based on these data.

### General distribution

The overall performance of intestinal I/R over the last 19 years was highly volatile, with the number of publications maximizing in 2020 and with a high publication profile in the previous two years. In contrast, the frequency of citations increased and tended to accelerate recently. The publication number and citation frequency can broadly describe the development of a field (Figure 1). We conclude that intestinal I/R, a long-established topic since the mid-20th century, has kept active in the first 19 years of the 21st century because it remains a severe challenge for physicians and patients in clinical practice.

China and the United States are the top two countries ranked by the number of publications and frequency of citations in intestinal I/R (Table 2). In terms of centrality and strength of association, the United States has the most decisive influence and vital linkage in the field. Spain and Italy have smaller numbers of publications but enjoy a high impact in the field, indicating their research results are generally considered as high-quality. Other high-impact countries are China, Germany, and England (Supplementary Figure 3). As the country with the most significant number of publications, China is also influential. However, the number of publications does not match with the centrality of other high-impact countries. In terms of the average frequency of citations per article, China (18.22) is still at a deficit position compared to other influential countries, such as the United States (37.46), Germany at (26.45), and England (22.2), and is even below the average level of 22.06 too many. There is a difference of about 1589 times in catching up with the world average, and the 4th most cited country exceeds the total citation frequency of Turkey. To catch up with the average citation level of the United States, it takes about 7,965 citations to exceed its citation frequency. However, contrary to the view at the national perspective, as far as institutions are concerned, China has 5 of the top 6 institutions in publications, and 4 of the top 10 institutions in citations ranked by association intensity, the first of which is Sun Yat-sen University in Guangdong, China (Table 2). The lack of national influence and the differences in Chinese dominance among institutions suggest that Chinese authors need to actively become international and improve their research quality and reference value. Moreover, there is a shortage of Chinese research in intestinal I/R that is good enough to be recognized and evaluated by the world. Similarly, we should recognize that international and inter-institutional cooperation in intestinal I/R is lacking (Figure 2B and Supplementary Figure 3). In addition to the apparent subgroups, no institution has a centrality over 0.1. We suggest that the field of intestinal I/R should strengthen cross-discipline and actively organize international conferences to attract researchers from more countries and institutions. And scientists should also apply basic research in the field of intestinal I/R to clinical trials and attract more doctors from hospitals to strengthen the links between institutions. Furthermore, Dalian Medical University has no links to other institutions (Supplementary Figure 4). When national and institutional barriers against communication can be broken down, the advantages between influential countries complement each other, and the platform for research is expanded, which will bring obvious benefits for the long-term progress of research in the field.

Supported by the data, Liu Kx has been the most prolific author in the field of intestinal I/R in the last 19 years, and early methodological and mechanistic discussions covered oxidative stress (228 citations, 7 articles), ischemia pre/post-conditioning (236 citations, 7 articles), and anesthetic drugs (318 citations, 5 articles). The most cited author is Chiu Cj, whose pioneering Chiu’s Score for intestinal injury assessment is widely used to judge the quality of intestinal I/R studies. Mallick Ih is the most cited author directly related to research in this field, with 535 citations for his Summary of Protective Strategies for Intestinal I/R Injury in 2004, followed by two gastroenterology conferences in 2005 reporting and describing the research and potential value of ischemia preconditioning in intestinal I/R. The results of his work on microcirculation in the 2000s (105 citations, 4 articles) are also an essential inspiration and background for subsequent research on intestinal I/R (Table 3). In addition, Tian Xf is the most cited author, with five studies on oxidative stress reaching 382 citations before 2016. A single article involving ferroptosis-induced intestinal injury in 2019 was cited 199 times recently. The scholars mentioned above have undoubtedly significantly contributed to the development of this field. Interestingly, Chiu Cj, as the most co-cited author, is not a researcher in the field, which is odd. In addition, we find 3 of the top 10 co-cited authors are from Louisiana State University, which may be an important institution for research in the field.

Reviewing the top 20 journals in terms of publication volume or co-citation frequency (Table 4), we find most of them are clinical journals, such as Journal of Surgical Research, World Journal of Gastroenterology, Transplantation Proceedings, and Gastroenterology. Some are essential medical journals, such as Journal of Immunology, which support and complete one of our inferences in “Distribution of Subject Areas” (Figure 6A). In other words, intestinal I/R is mainly clinical medical research to complement basic medical research. The two are mutually supportive and translatable. We obtained the impact factor (IF) and quartile (Q) of the published and cited journals on intestinal I/R in JCR 2020. The top three published journals are Journal of Surgical Research (IF 2.192, Q3), Shock (IF 3.454, Q2), and World Journal of Gastroenterology (IF 5.742, Q2). Only World Journal of Gastroenterology division is higher than Q3, and IF is not lower than 5. The results in the field need to be improved in terms of quality. In conclusion, most of the journals in this field have IF higher than World Journal of Gastroenterology (IF 5.742) and Journal of Immunology (IF 5.422), and the highest IF among the frequently cited journals is Gastroenterology (IF 22.682). We clustered the citation relationships of the journals, and found frequent associations among journals within subgroups, but not as apparent as associations among institutions or authors (Figure 5), which is partly because the authors maintained good academic habits and read and thought widely about results at different levels.

### Hotspots and frontiers

Keyword analysis is beneficial to grasping the core contents and frontiers of a research field (51). In the existing studies, the main keywords are “intestine”, “oxidative stress”, “inflammation”, “apoptosis”, “lung injury”, “rat”, “cytokine”, “nitric oxide”, “antioxidant” and “bacterial translocation” (Table 6). These keywords and their relationships are often closely associated with the research hotspots regarding intestinal I/R process mechanisms, such as the relationship among inflammation, intestinal barrier and vascular permeability. The manifestations are in-situ organ and distant organ damages and the pathway mechanisms of oxidative stress. Intestinal I/R therapies include ischemic pre/post-treatment, hydrogen resuscitation, transcriptional inhibition, and related drugs such as anesthetic drugs (Figure 8). However, deriving of the above hotspots still need years of keyword data support, and the timeliness is yet unsatisfactory. We unfolded the analysis through the time of highly explosive keywords. In recent years, lung injury and various cell death such as apoptosis, autophagy, and ferroptosis caused by intestinal I/R are also worthy of attention. Despite the lack of explosive trend in flora, exosomes, or TLR, there are a certain number of studies on discussion of intestinal I/R in the last three years.

The cell death modalities currently explored in intestinal I/R are apoptosis, pyroptosis, ferroptosis, autophagy, NETosis, and necrotizing apoptosis. In the last few years, metformin, which is used extensively to study autophagy, has been discussed in intestinal I/R for its possible involvement in pyroptosis via the TXNIP-NLRP3-GSDMD pathway (52). Research on autophagy in intestinal I/R shows multiple signaling pathways play a role. For instance, paeoniflorin activation of LKB1/AMPK pathway and mTOR signaling is positively correlated with, and the JAK2/STAT3 signaling pathway is negatively associated with the occurrence of autophagy (53–55). Some pathways may exhibit other effects while promoting autophagy. Post-ischemic treatment through the Akt/GSK-3β/Nrf2 pathway can inhibit oxidative stress and make autophagy accompanied by positive antioxidant effects (56). The transcription factor Nrf2 in its pathway may be involved in multiple cell death modalities, including apoptosis. In intestinal I/R, Nrf2 plays a protective role as it can regulate SLCA11 and HO-1 to limit ferroptosis to attenuate secondary acute lung injury, and can modulate TERT and SLC7A11 to weaken secondary acute liver injury (57). With oxidative stress imbalance as an essential death phenotype, ferroptosis has an oxidative stress-related upstream similar to Nrf2. ACSL4 was first identified in intestinal I/R to play a role in ferroptosis-mediated injury via ischemia production (41). Administration of apigenin-7-O-β-d-(-6″-p-coumaroyl)-glucopyranoside prevented ferroptosis in intestinal vascular endothelial cells by inhibiting the downstream Nrf2 of HO-1 and MAO-B (58). Intriguingly, inhibitors of the apoptosis-associated protein p53 similarly inhibited ferroptosis in intestinal I/R (42). Furthermore, SIRT3-mediated deacetylation of PRDX3 attenuated intestinal I/R apoptosis while alleviating oxidative mitochondrial damage (59). In contrast, mitochondrial autophagy in a diabetic mouse model exacerbated intestinal I/R damage (60), while the miR-665-3p/ATG4B/autophagy regulatory axis had anti-inflammatory and anti-apoptotic effects (61). All these shreds of evidence implicitly and inconspicuously point to the possible correlation and mutual checks and balances among apoptosis, autophagy, and ferroptosis in intestinal I/R. Interestingly, a similar concept has recently been proposed for PANoptosis, whereby cell-bound inflammatory cytokines initiate a cell death process that is highly interconnected with pyroptosis, apoptosis and necroptosis through signaling induced by death-containing structural domains of receptors (62, 63). Two recent discoveries are that NETosis is also involved in the development of intestinal I/R along with necrotic apoptosis, and that HMGB-1, a DAMP, can induce NETosis and thus exacerbate acute lung injury induced by intestinal I/R (64). Surprisingly, bacterial invasion can reduce the NETosis-caused damage (65). Recently, how thrombus recombinant protein, a neutralizer of NETs, attenuates NETosis in intestinal I/R was investigated (66). Reports on necroptosis show that mtDNA-STING is involved in necrosis of the intestinal epithelium following intestinal I/R (67). The livers will be secondary to HMGB-1-associated necrosis and polarization of Kupffer cell M1 (68).

Given that intestinal I/R occurs surgically on patients under anesthesia, the possible effects of anesthetic drugs and their resulting individual mental status on intestinal I/R shall be evaluated. There are many relevant studies recently, which cover the medications of propofol, dexmedetomidine, etomidate, remifentanil, and sevoflurane. Propofol, a general anesthetic, is seemingly protective in intestinal I/R through PI3K/Akt and NF-κB pathways (69, 70). Similarly, etomidate and sevoflurane may have NF-κB protective pathways in intestinal I/R (71, 72). In contrast, remifentanil promotes PDIA3 expression by activating p38MAPK to inhibit oxidative and endoplasmic reticulum stress in intestinal I/R (73). Dexmedetomidine, commonly used as an anesthetic sedative, is the most carefully-studied anesthetic drug in terms of the effects on intestinal I/R. As for intestinal injury mechanisms, dexmedetomidine may inhibit p38MAPK, and TLR4/MyD88/NF-κB signaling pathways and activate Jak/STAT signaling pathways, attenuating intestinal injury (74–76). Reportedly, dexmedetomidine pretreatment alleviates intestinal vascular barrier injury and is clarified to attenuate distal liver injury (77).

Furthermore, the hepatoprotective effect of dexmedetomidine was enhanced by the combination of irisin (78). The protective effect of dexmedetomidine on acute lung injury, a hot topic at this stage, was recently discussed in a rat model (79). Dexmedetomidine can protect acute lung injury probably because inhibition of NLRP3 inflammatory vesicles in lung tissues or the activation of PI3K/Akt leads to the upregulation of cannabinoid receptor 2 and subsequent effects (80). In addition to total organ damage, other research topics of dexmedetomidine are mitochondria-related oxidative stress and endoplasmic reticulum stress. Dexmedetomidine promotes mitochondrial translocation of telomerase reverse transcriptase, protecting enteric glial cells and reducing oxidative stress from intestinal injury (81). The action mechanism of dexmedetomidine on mitochondria is strongly related to the SIRT family, which inhibits endoplasmic reticulum stress-induced pyroptosis in intestinal epithelial cells via SIRT1 expression (82). Moreover, the SIRT3-dependent regulation of the PINK1/HDAC3/p53 pathway can inhibit mitochondrial damage and apoptosis in enteric glial cells (83).

In a very complex microbial environment, the intestine maintains a dynamic equilibrium through mucosal and other barriers to interact with the flora, which is highly susceptible to disruption by intestinal I/R. The role of the intestinal flora and metabolites in intestinal I/R has attracted increasing attention recently. Flora can attenuate NETosis in acute mucosal injury in intestinal I/R (65). Preoperative fasting by mimicking clinical requirements for patients was performed before the mouse model of intestinal I/R, and its alteration of flora metabolites was found beneficial for resisting intestinal I/R injury (84). Specific flora such as Bifidobacterium bifidum PRL2010 can attenuate intestinal I/R injury (85), and altered schaedler flora affected leukocyte adhesion in intestinal I/R injury (86). In terms of specific mechanisms, the flora metabolite pravastatin drives IL-13 release from type II innate lymphocytes via IL-33/ST2 signaling and attenuates intestinal I/R injury (87). Capsiate activates TRPV1 and Gpx4 expression, and inhibits intestinal I/R-induced ferroptosis (15).

Other frontiers of our focus are exosome and TLR-related studies. Current technology allows the isolation of intestine-derived exosomes in the mouse intestinal I/R model, which facilitates subsequent studies (88). Intestine-derived exosomes can stimulate microglia and affect memory deficits after intestinal I/R (89). Classically, the main components of exosomes are believed to contain miRNAs, among which miRNA-26b-5p can target DAPK1 to inhibit apoptosis of intestinal mucosal cells in intestinal I/R (90). miR-665-3p can attenuate inflammation and apoptosis in intestinal I/R via the ATG4B-autophagy regulatory axis (61). miR-34a-5p activation of SIRT1 signaling attenuates reactive oxygen species accumulation and apoptosis (46). Therefore, the protection of intestinal I/R by intestine-derived exosomes may be a new potential therapy for intestinal I/R injury. In addition, TLR, with 11 family members, recognizes PAMP receptors and equally recognizes DAMP receptors. The role and mechanism of TLR4 in intestinal I/R injury are widely studied and reported (91). More scholars start to focus on the parts and tools of other TLR members in intestinal I/R. In terms of PAMP receptors, Lactobacillus murinus can promote IL-10 release from M2 macrophages through activation of TLR2 signaling, thereby attenuating intestinal I/R injury (16). In terms of DAMP receptors, intestinal I/R leads to increased expression of extracellular RNA, which is further a potential risk molecule for activating TLR3 to trigger or even exacerbate intestinal I/R injury (92).

## Limitations

We statistically analyzed the trends, hot spots, and frontiers in intestinal I/R over the last 20 years by using three software programs: CiteSpace, VOSviewer, and R-bibliometrix. The discussion in this review still has many limitations. All literature data here are only from Web of Science. Although the timely and dynamic updating, authority, and extensiveness of the Web of Science helped us to obtain abundant literature to support the analysis, we cannot exclude the possibility of leaving out a part of the results about intestinal I/R that other data engines cannot ignore. In addition, our analysis relies heavily on the accumulation of literature data over time and the homogeneity within keywords. In consideration of analysis softwares have not enough to hadle the defect of data, we analyzed the most recent period, and screened some different results. However, we still unavoidably missed some frontier hot spots in intestinal I/R.

## Conclusion

In this study, we reviewed the 19 years of research on intestinal I/R using bibliometric analysis, and analyzed the development of this field in space-time, internal logic, current situation, hot spots, and frontiers from multiple perspectives. Undoubtedly, the United States and China are the core competencies of intestinal I/R research. However, there is an extraordinary lack of communication between the two sides and even among the possible subgroups, institutions and authors. Among all the authors, Liu, Kexuan has the largest number of articles and great influence in the field of intestinal I/R. Chiu, Cj was most co-cited by the intestinal I/R field researchers for the important role of Chiu’s Score in the evaluation of intestinal injury. Journal of Surgery Research is the most important journal to intestinal I/R as the hugest size both in Publications and Citations. Specifically, the mechanisms of tissue-bacteria interactions, the association between modes of death, and the role of enteric exosomes or clinical anesthetic drugs will significantly determine the results of these studies in intestinal I/R. In summary, our results showed a comprehensive bibliometric analysis of research in intestinal I/R from a global perspective and may provide helpful clues for future research directions and scientific decision-making in this domain.

## Data availability statement

The original contributions presented in this study are included in the article/Supplementary material, further inquiries can be directed to the corresponding author/s.

## Author contributions

XYZ and KL: study conception. YW, PD, and XBZ: study design. YW, PD, XBZ, YL, and JS: study conduct. YW: data analysis. YW, PD, and XBZ: full access to all the data in the study, take responsibility for the integrity of the data and the accuracy of the data analysis, data interpretation, and drafting of the manuscript. YW, PD, XBZ, YL, JS, WL, KL, and XYZ: critical revision of the manuscript for important intellectual content. All authors contributed to the article and approved the submitted version.
