# Supplementary material for: Bibliometric and visual analysis of intestinal ischemia reperfusion from 2004 to 2022
Source: Front Med (Lausanne). 2022 Aug 15;9:963104. doi: 10.3389/fmed.2022.963104 (PMC9426633; doi:10.3389/fmed.2022.963104)
Supplement: Supplementary file 1 [file Data_Sheet_1.docx]

Supplementary Material

# Supplementary Data

The direct citation relationship between the highly-cited literature and its temporal distribution was observed (Supplementary Figure 1)(48). Two highly-cited articles started in 2004. However, no excessive direct citation relationship was found between the high-cited articles before and after 2007. Even the article by El-assal in 2005 and the article by Rocourt Dv in 2007, except for the relationship between them, have no direct citation relationship with other high-cited articles. Frequent discussion between the highly-rated articles started in 2008. The most frequently referenced high-cited articles are the three articles from Liu Kx in 2007-2009(49-51), and the two articles from Grootjans J in 2010 and 2011(52, 53), who both have high academic reputation and prestige in the field. The more highly-cited articles with more references are those from Li Ys (17)and Wen Sh (54)in 2017 and from the respective teams of Nadatani Y (55)and Li Zl (56)in 2018.

In addition to the longitudinal time observation, some traces of the research ideas can also be found in the interaction of several horizontal periods(60). A surge in emerging keywords occurred around 2010. A significant part of the overall thinking was translated from nf-kappa b, apoptosis, inflammation, and injury (2004 - 2010) to inflammation (2011 - 2018), and ultimately to inflammation, colic, and dexmedetomidine (2019 - 2022). The recent noteworthy inos was more influenced by nitric oxide from 2011 - 2018 and il-1beta and melatonin from 2004 - 2010. The antioxidant was maintained in many studies since 2011, and was partly derived from the study on nf-kappa b before 2010. Recent studies on sepsis and acute respiratory distress syndrome were influenced by the shock study in 2011 - 2018 and may be related to the results of inflammation and endothelium in 2004 and 2010. The endothelium is closely related to sepsis through shock and microcirculation from 2011 to 2018 (Supplementary Figure 2A). However, not all the studies continued. After the “injury” burst in 2007-2009 to 2018, the research in 2018-2020 focused on acute mesenteric ischemia, which seemed to be a brief and intense hotspot that was not at the forefront (Supplementary Figure 2B). We can classify the keywords into two fields, including clinical medicine (surgery, pediatrics, hepatology, neuroscience, critical care medicine, anesthesia), and non-clinical medicine (immunology, physiology, biochemistry, molecular biology, pharmacy, pharmacology, and biophysics). We recognized such a research process from clinical to basic medical research and back to clinic research. From 2004 to 2009, there were mainly clinical studies with the keywords inflammation, bacterial translocation, intestinal ischemia, hypothermia, and melatonin. In 2010 - 2018, the leading research was on basic medicine, with the keywords glutamine, hydrogen-rich saline, complement, antioxidant and histopathology (2010 - 2012), tumor necrosis factor-alpha, antioxidant, malondialdehyde and hydrogen (2013 - 2015), liver injury, intestinal injury and mesenteric ischemia (2016 - 2018). In 2019 - 2022, the research was refocused on the clinic. In addition to inflammation and intestinal ischemia, the new keywords were sepsis, colic, propofol, and autophagy (Supplementary Figure 2C).

Citespace, developed by Chaomei Chen of Drexel University, USA, are widely popular, which draw mappings with large amounts of information and good visual effect and can provide scientific perspectives from different sides. To proof the Figure 3,4 by Vosviewer, Supplementary Figure 3,4 are made by CiteSpace.

# Supplementary Figures and Tables

## Supplementary Figures


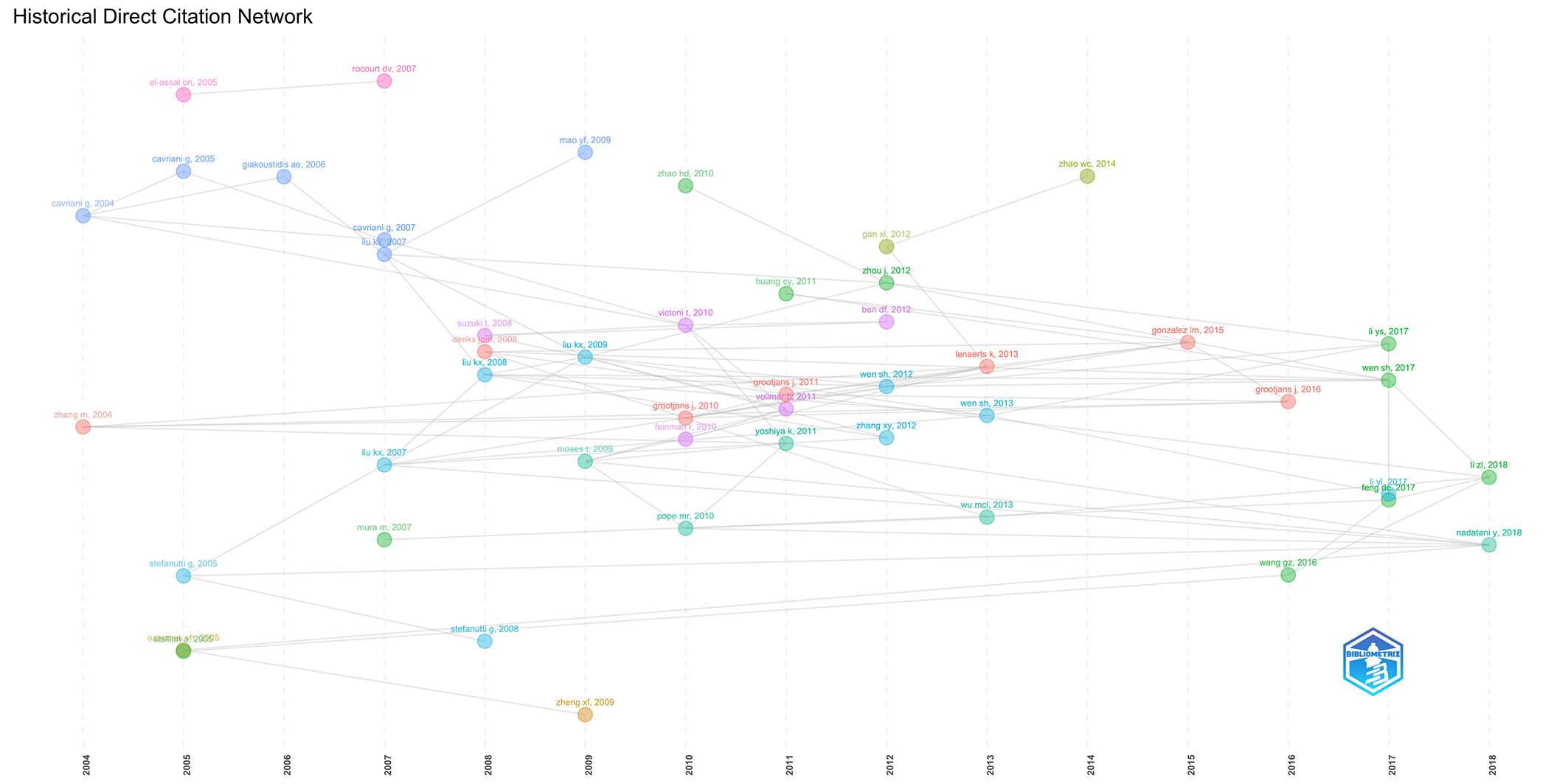


**Supplementary Figure 1.** Temporal distribution and citation relationships of highly cited literature.


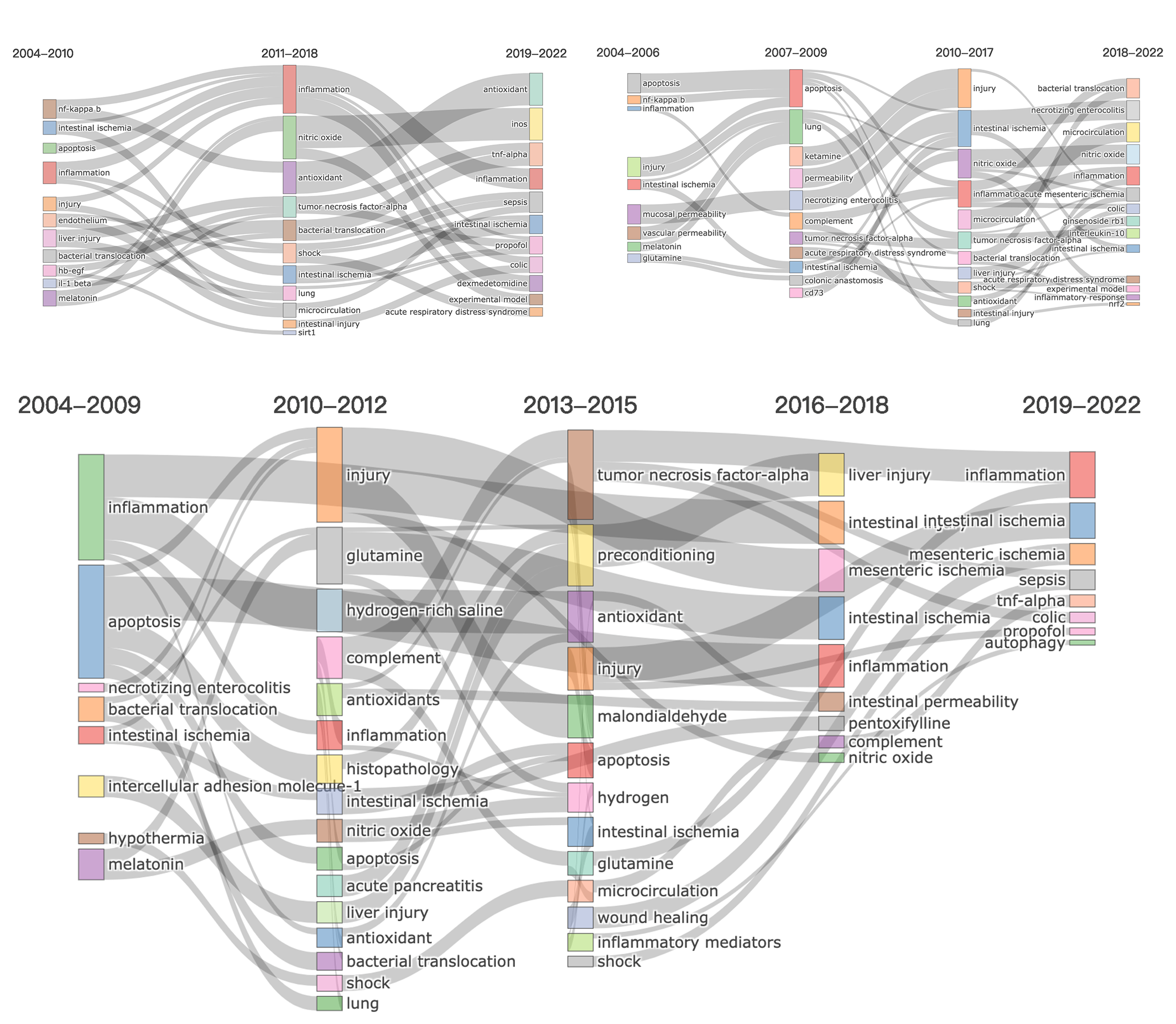


**Supplementary Figure 2.** By segmenting the different refinements in time, a **(A)** three-segment map, **(B)** four-segment map, and **(C)** five-segment map were obtained for the total keyword time tracing.


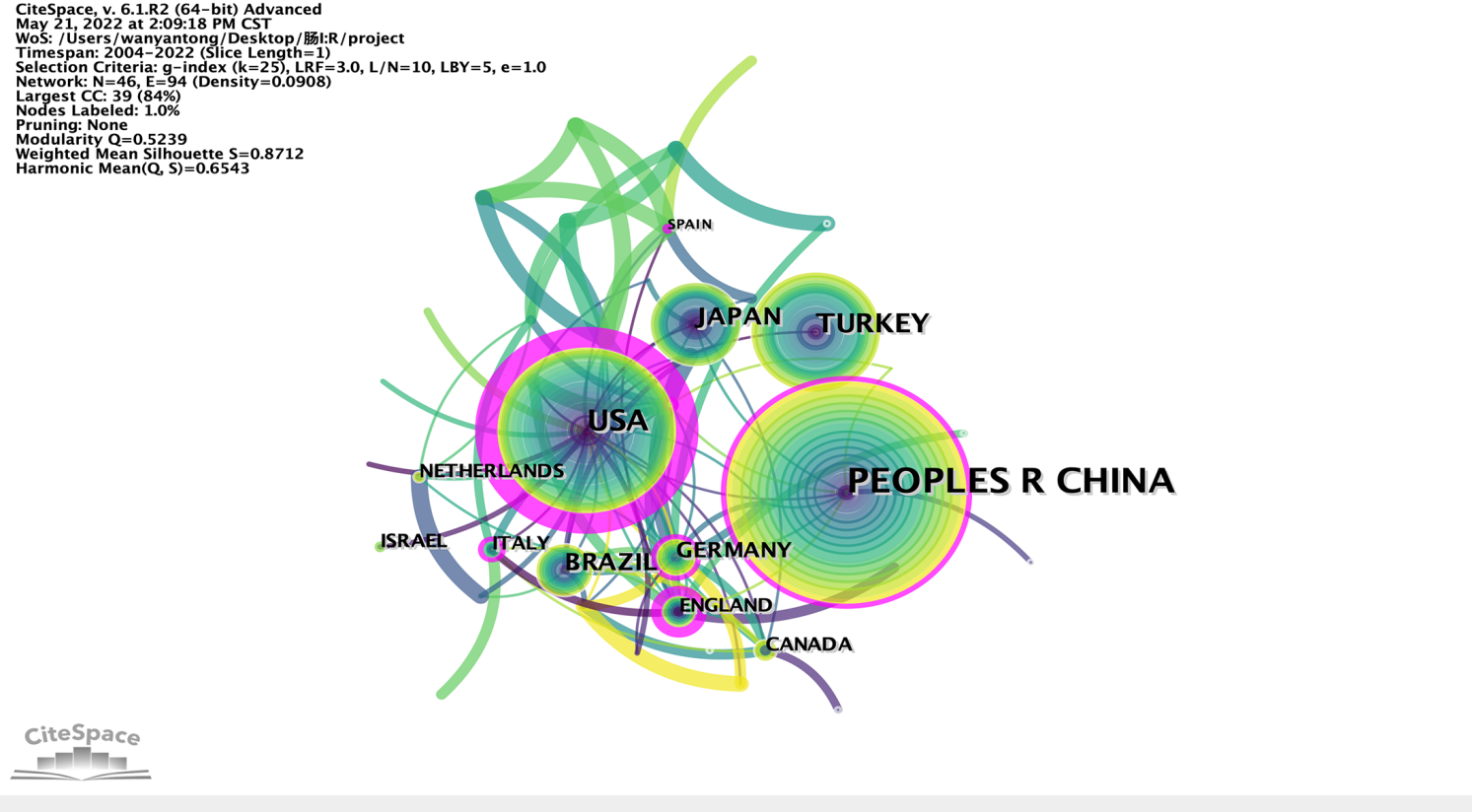


**Supplementary Figure 3.** Applying CiteSpace, the collaborative network of countries/regions. The thickness of the connected lines indicates the strength of the relationship between the nodes.


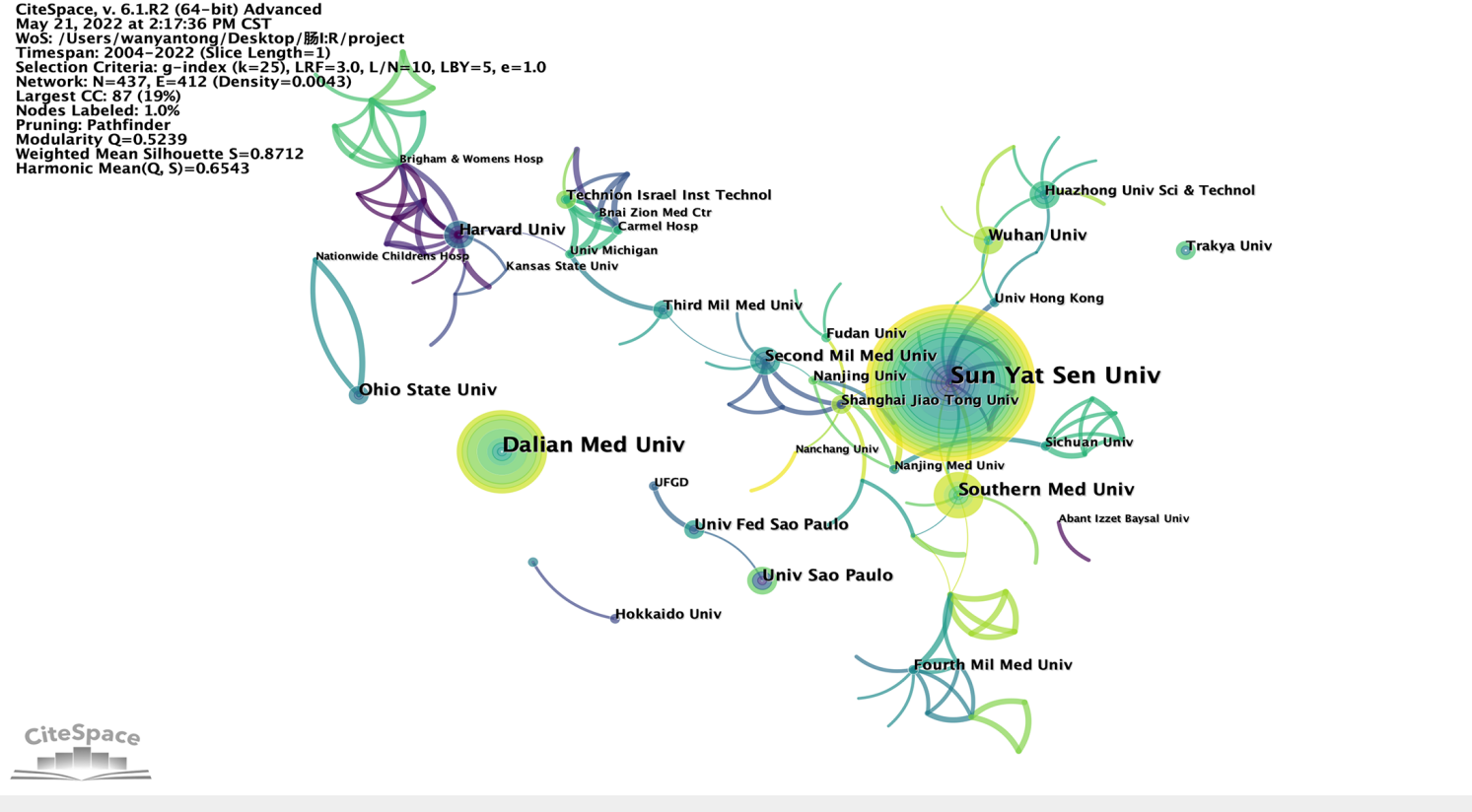


**Supplementary Figure 4.** Applying CiteSpace, the collaborative network of institutions. The thickness of the connected lines indicates the strength of the relationship between the nodes.
